# Supplementary material for: Performance of Convolutional Neural Networks for Polyp Localization on Public Colonoscopy Image Datasets
Source: Diagnostics (Basel). 2022 Apr 4;12(4):898. doi: 10.3390/diagnostics12040898 (PMC9027927; doi:10.3390/diagnostics12040898)
Supplement: Supplementary file 1 [file diagnostics-12-00898-s001.zip › diagnostics-1630815-SM.pdf]

Table S1. Summary of datasets' structure and conversion process statistics.

Dataset	Folder structure	Image format	Ground truth format	No. of files	No. of bounding boxes	Average bounding box area (relative to image area)	Format conversion script	Folder organization script	Dataset conversion script
CVC-ClinicDB	├── bbdd<br>├── border<br>├── gtlumen<br>├── gtpolyp<br>└── gtspecular	BMP	TIFF	612	670	0.13	yes		CVC-ToVOC.py
CVC-ColonDB	├── bbdd<br>├── border<br>├── gtlumen<br>├── gtpolyp<br>└── gtspecular	BMP	BMP	300	300	0.08	yes		CVC-ToVOC.py
CVC-PolypHD	└── SegmentationTrainingUpload	BMP	TIFF	56	64	0.10	yes	separete_folder_<br>PolypHD.sh	PolypHDToVOC.py
ETIS-Larib	├── ETIS-LaribPolypDB<br>└── Ground Truth	TIFF	TIFF	196	208	0.06	yes		ETIS-LaribToVOC.py
Kvasir-SEG	├── images<br>├── kavsir_bboxes.json<br>└── masks	JPG	JPG	1 000	1 071	0.21	no		KvasirToVOC.py
CVC-ClinicVideoDB	├── cvcvideoclinicdbtestpart1.rar<br>├── cvcvideoclinicdbtestpart2.rar<br>└── CVC-VideoClinicDBtrain_valid<br>└── *	PNG	PNG	11 954	10 025	0.06	yes	separate_folder_<br>ClinicVideo.sh	ClinicVideoToVOC.py
PICCOLO	├── test<br>│ ├── masks<br>│ ├── polyps<br>│ └── void<br>├── train<br>│ ├── mask<br>│ ├── polyps<br>│ └── void<br>└── validation<br>├── mask<br>├── polyps<br>└── void	TIFF	PNG	3 433	4 026	0.23	yes	merge_PICCOLO.sh	PICCOLOToVOC.py
KUMC dataset	├── test2019<br>│ ├── Annotation*<br>│ └── Image*<br>├── train2019<br>│ ├── Annotation<br>│ └── Image<br>└── val2019<br>├── Annotation*<br>└── Image*	JPG	XML	38 697	36 773	0.08	no		KUMCToVOC.sh
SUN	├── sundatabase_positive_part1.zip<br>└── sundatabase_positive_part2.zip	JPG	TXT	49 136	49 136	0.12	no	merge_SUN.sh	SUNToVOC.py
LDPolypVideo	├── Test<br>│ ├── Annotations*<br>│ └── Images*<br>└── TrainValid<br>├── Annotations*<br>└── Images*	JPG	TXT	40 187	37 632	0.06	no	merge_and_rename_<br>LDPolypVideo.sh	LDPolypVideoToVOC.py

*Group by polyp
